# The cancer‐associated fibroblasts related gene CALD1 is a prognostic biomarker and correlated with immune infiltration in bladder cancer

**DOI:** 10.1186/s12935-021-01896-x

**Published:** 2021-05-29

**Authors:** YiHeng Du, Xiang Jiang, Bo Wang, Jin Cao, Yi Wang, Jiang Yu, XiZhi Wang, HaiTao Liu

**Affiliations:** 1 Department of Urology, Suzhou Kowloon Hospital, Shanghai Jiaotong University School of Medicine, 215028 Suzhou, China; 2 Department of Pathology, Suzhou Kowloon Hospital, Shanghai Jiaotong University School of Medicine, 215028 Suzhou, China; 3 Department of Urology, Shanghai General Hospital, Shanghai Jiaotong University School of Medicine (originally named “Shanghai First Hospital”), 200080 Shanghai, China

**Keywords:** Bladder cancer, Cancer‐associated fibroblasts, CALD1, Immunosuppression, Prognostic biomarker

## Abstract

**Background:**

Stromal components of the tumor microenvironment contribute to bladder cancer progression, and Cancer-Associated Fibroblasts (CAFs) were reported to play an important role. Accumulating pieces of evidence indicate that CAFs participate in the crosstalk with tumor cells and have a complex interaction network with immune components. Further studies on the role of CAFs in the bladder cancer microenvironment and searching for possible specific markers are important for a deeper understanding of CAFs in bladder cancer progression and immunomodulation.

**Methods:**

In the present study, we examined the abundance of CAFs in the TCGA and GEO datasets using the MCP-COUNTER algorithm. Additionally, the expression of genes related to CAFs was analyzed through the Weighted Gene Co-expression Network Analysis (WGCNA). The CIBERSORT and ESTIMATE algorithms were used to discuss the correlation of the key CAFs-related gene and the tumor microenvironment components. Immunohistochemistry analysis in clinical samples was used to validate the results of bioinformatics analysis.

**Results:**

The results showed that CAFs were closely associated with the progression and prognosis of bladder cancer. WGCNA also revealed that CALD1 was a key CAFs-related gene in bladder cancer. Moreover, further in-depth analysis showed that CALD1 significantly affected the progression and prognosis of bladder cancer. The CIBERSORT and ESTIMATE algorithms demonstrated significant correlations between CALD1 and the tumor microenvironment components, including CAFs, macrophages, T cells, and multiple immune checkpoint related genes. Finally, immunohistochemistry results validated the strong association of CALD1 with CAFs and macrophages.

**Conclusions:**

In the present study, we confirmed the cancer-promoting roles of CAFs in bladder cancer. Being a key gene associated with CAFs, CALD1 may promote bladder cancer progression by remodeling the tumor microenvironment. The bioinformatics methods, including the CIBERSORT, MCP-COUNTER and ESTIMATE algorithms, may provide important value for studying the tumor microenvironment.

## Introduction

Bladder Cancer (BLCA) is one of the most common malignant tumors of the genitourinary system [1]. Based on the invasion depth, BLCA can be divided into Non-muscle Invasive (NMIBC) and Muscle Invasive Bladder Cancer (MIBC). Notably, NMIBC has a better prognosis and more treatment options compared to MIBC [2]. Besides, intravesical instillation of Bacillus Calmette-Guerin (BCG) was first reported to be effective in treating NMIBC by Morales et al. in 1976, with complete remission rates up to 70−80 % [3]. This was the earliest application of immunotherapy for the management of BLCA and has been used up to date. Moreover, BLCA represents a growing number of cancers characterized by the infiltration of a significant number of immune cells in the tumor microenvironment (TME) [4, 5], making it suitable for immunotherapy.

Tumorigenesis and tumor development are complex processes affected by many factors. In addition to genetic changes and epigenetic defects. The TME, which consists of cellular and non-cellular components, also plays an essential regulatory role [6]. Tumor stromal cells are the main cellular components of the TME. Furthermore, stromal cells in the TME, especially Cancer-Associated Fibroblasts (CAFs) [7], have multiple effects on cancer growth and maintenance.

CAFs interact with immune components by secreting various factors such as collagen, Matrix Metalloproteinases (MMPs), and chemokines [8]. Additionally, Tumor-Infiltrating Immune Cells (TIICs) in the TME were critically associated with cancer outcomes. Out of all the TIICs, macrophages are always among the most abundant in the TME, including in BLCA [9]. Moreover, existing evidence indicates that CAFs interact with the M2 macrophages, referred to as Tumor-Associated Macrophages (TAMs), promoting immunosuppression and inducing the occurrence and progression of cancers [10].

Furthermore, bioinformatics methods are now widely used in cancer research. Through high-throughput sequencing, mechanisms underlying pathological processes, including cancers, can be revealed by comparing different genes’ expression networks. Currently, the CIBERSORT [11] and MCP-COUNTER [12] algorithms for calculating the abundance of immune cells are widely used in studies of the TME. CAFs scores and relative levels of 22 TIICs can be computed using the MCP-COUNTER and CIBERSORT algorithms, which provide significant help in studying the relationship between CAFs and TIICs. Moreover, genes highly co-expressed in cancer can be identified through the Weighted Gene Co-expression Network Analysis (WGCNA) [13]. Therefore, through these bioinformatics means, the present study uncovered and validated that Caldesmon 1 (CALD1), a key gene associated with CAFs, was crucial in regulating both the stromal and immune microenvironment of BLCA. Consequently, it may become a promising biomarker of BLCA progression.

## Methods and materials

### The source of data

The gene expression quantification data for transcriptome profiling included 430 samples (19 normal samples and 411 tumor samples) from 405 bladder transitional cell carcinoma patients. All the data was downloaded from the TCGA database (https://portal.gdc.cancer.gov/). For samples from the same patients, gene expression was made average by the Bioconductor *limma* package of R software (version 4.0.2). Moreover, the gene expression profiling dataset (GES13507) was downloaded from the Gene expression Omnibus (https://www.ncbi.nlm.nih.gov/geo/), including 165 primary BLCA samples, 23 recurrent non-muscle invasive tumor tissues, 58 normal looking bladder mucosae surrounding Cancer, and the corresponding clinical data.

### Calculation of CAFs scores

The MCP-counter algorithm [12, 14], provided by TIMER 2.0 (http://timer.cistrome.org/), was used to calculate the CAFs score of patients recruited from both the TCGA and GEO databases.

### Estimation of the tumor microenvironment

The ESTIMATE algorithm in the estimate package of the R software was used. Three scoring forms, namely immune score, stromal score and ESTIMATE score, were positively correlated with the proportion of immune and stromal components as well as the sum of both. Therefore, it is indicated that the higher the score, the more significant the ratio of the corresponding parts in the TME.

### Screening for differentially expressed genes (DEGs)

To screen for DEGs in both the TCGA and GEO cohorts, we divided the samples into two groups (High and low CAFs) according to the medium level of the CAFs score. After that, analysis of differential gene expression between these two groups was performed using the Bioconductor *limma* package of the R software. Genes with adjusted p-value < 0.05 and |log_2_FC|>1 were considered to be significantly differentially expressed.

### WCGNA for the determination of key genes

The Weighted Gene Correlation Network Analysis (WGCNA) R-package was used for co-expression network analysis. CAFs related genes were selected from the most significant modules related to high levels of CAFs. Hub genes were obtained from the intersection of CAFs related genes and DEGs in both the TCGA and GEO cohorts.

### GO and KEGG enrichment analysis

The “clusterprofiler” R package was used to perform GO and KEGG enrichment analyses for hub genes. GO terms or KEGG pathways with corrected P-value < 0.05 were considered to be significantly enriched.

### Gene set enrichment analysis (GSEA)

The Hallmark and C2 Kegg gene sets v7.2 were used for GESA, which was performed using the GSEA software (version 4.1.0) obtained from the Broad Institute. Gene sets with NOM p < 0.05 and False Discovery Rate (FDR) q < 0.05 were considered to be significant.

### Survival analysis

Survival analysis was conducted using the “survival” and “survminer” packages of the R software. Additionally, the Kaplan-Meier method with the best cut-off value was used to draw the survival curves. P values from the log-rank test that was less than 0.05 were considered to be significant.

### TIICs Profile

The R software’s CIBERSORT algorithm was used to determine the profile of TIICs (including 22 immune cells) in all tumor samples of the TCGA cohort. 235 patients with p < 0.05 were selected for subsequent analysis.

### Patients recruitment

40 BLCA specimens from the patients of Shanghai General Hospital were selected, including 27 male and 13 female with different stage, TNM classification. The brief information of these patients was listed in the following Table 1.

**Table 1:** Brief information of the 40 patients recruited for IHC analysis

Variable	Age		Grade		Stage		T				N		M	
	<70	≥ 70	High grade	Low grade	I–II	III–IV	T1	T2	T3	T4	N0	N1	M0	M1
N	21	19	29	11	35	5	22	13	3	2	37	3	39	1
Male	18	9	19	8	23	4	14	9	2	2	25	2	26	1
Female	3	10	10	3	12	1	8	4	1	0	12	1	13	0

### Immunohistochemistry analysis

Here, we used ACTA2, a marker of CAFs, and CD206, a marker of macrophage M2, to discuss the relation between CALD1, CAFs, and macrophage M2. The expression of CALD1 (1:250, Abcam, ab32330), ACTA2 (1:250, Abcam, ab7817), and CD206 (1:4000, Abcam,ab252921) in tumor tissues was detected using the BenchMark GX automatic immunohistochemical staining system (Roche, Switzerland). After deparaffinization, the tissue sections were incubated with primary antibody for 32 min. Biotinylated anti-IgG antibody and horseradish peroxidase were used to show positive expression areas. Hematoxylin was used for counterstaining and Bluing Reagent for post counterstaining.

### Evaluation of the immunohistochemical results

Two pathologists (Xiang J & Jin C) evaluated the immunohistochemical results without prior acknowledgment of the patient’s clinical data and discussed any discrepancies in scores until a consensus was reached. IHC score was measured according to the staining intensity and the proportion of positive stromal cells. The standard was as followed: [IHC score 1], weak staining in < 50 % or moderate staining in < 20 % of stromal cells; [IHC score 2], weak staining in ≥ 50 %, moderate staining in 20–50 % or strong staining in < 20 %; [IHC score 3], moderate staining in ≥ 50 % or strong staining in ≥ 20 %. Cases with scores 2 or 3 were regarded as positive for each protein expression [15].

### Statistics analysis

Univariate Cox regression was performed using the “survival” package of the R-software. Genes with p values of less than 0.05 were considered to be related to survival. Moreover, survival-related hub genes were identified from the intersection of survival-related genes in the TCGA and GEO cohorts. Correlation analysis was conducted through the Pearson (normally distributed) and Spearman (abnormal distribution) correlation test. Gene expression was transformed by log2 (FPKM + 1). Differential analysis between the high and low CALD1 expression groups was then conducted through the Wilcoxon test. The Kruskal test estimated statistical significance for variables of more than two groups. The Fisher exact test was used to analyze the correlation of CALD with CD206 and ACTA2. P-value < 0.05 was considered significant.

## Results

### Abundance of CAFs is a poor prognostic factor associated with the progression of BLCA

**Fig. 1:**
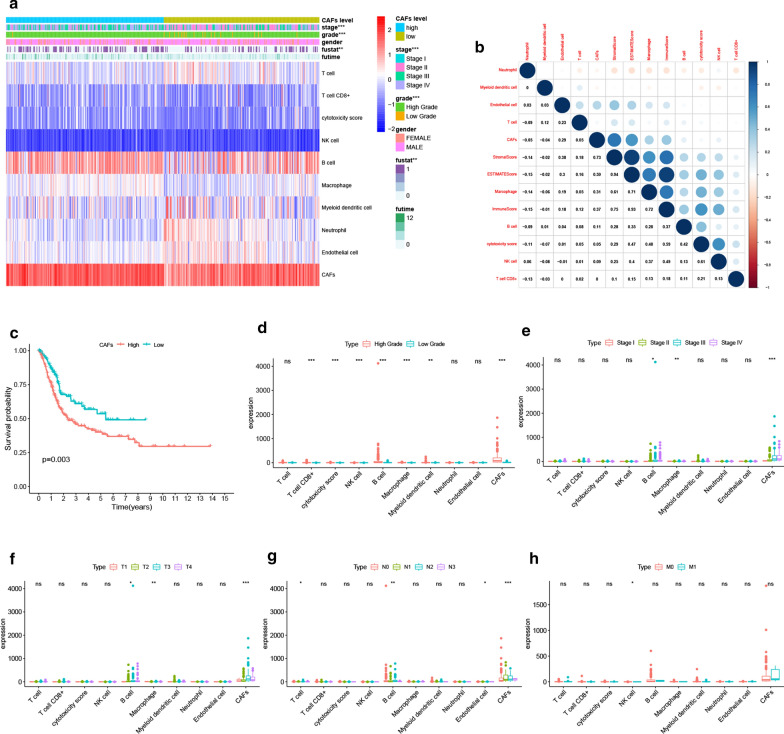
CAFs abundance affected BLCA progression in the TCGA cohort. **a** The abundance of different cell types calculated by MCP-COUNTER was shown in the heatmap. There were significant differences in tumor stage, grade and patient survival status between the high and low CAFs groups. **b** After the correlation analysis, close correlations of CAFs with stromal scores (R = 0.73), immune score (R = 0.37), and ESTIMATED score (R = 0.59) were observed. **c** Kaplan-Meier survival analysis demonstrated that patients with high CAFs abundance exhibited significant lower OS compared to low CAFs patients (p = 0.003). **d–h** Different CAFs abundance was observed regarding BLCA grade (p < 0.001), stage (p < 0.001), T (p < 0.001) and N (p < 0.001) classification

**Fig. 2:**
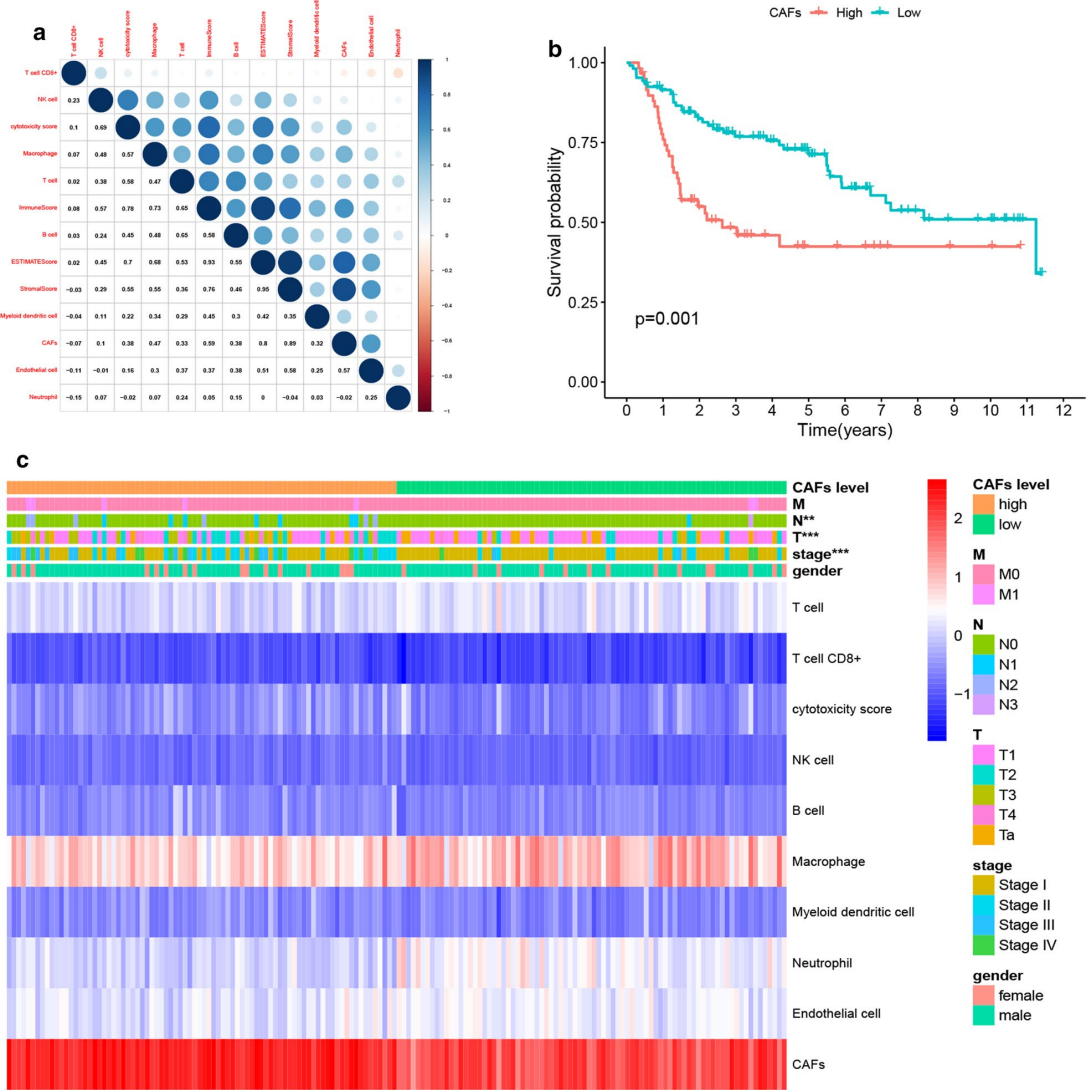
Validation of the influence of CAFs abundance on BLCA progression in GEO cohort. **a** The correlation of TME cell types with ESTIMATE scores in the GEO cohort. High correlations between CAFs, stromal score (R = 0.89), immune score (R = 0.59) and ESTIMATE score (R = 0.80) were also observed. **b** Kaplan-Meier survival curves of high vs. low CAFs abundance in patients of GEO cohort. Patients in the high CAFs group presented a significantly lowered OS (p = 0.001). **c** The expression of each TME cell was shown after grouping by CAFs. Significant differences were found in stage, T and N classification between CAFs low and high abundance groups

We first calculated the abundance of CAFs in the TCGA cohort using the MCP-COUNTER algorithm. The results indicated that CAFs were more abundant than any other cell types in the tumor microenvironment (Fig. 1a) and had a significant correlation with the stromal score (R = 0.73), immune score (R = 0.37) as well as the ESTIMATE score (R = 0.59) (Fig. 1b). Moreover, high levels of CAFs were significantly associated with low survival in BLCA patients, as shown in Fig. 1c (p = 0.003). Furthermore, we compared the effects of different cell types in the microenvironment on the clinical characteristics of BLCA. Notably, CAFs were found to have a significant impact on BLCA grade as shown in Fig. 1d (p < 0.001), stage as highlighted in Fig. 1e (p < 0.001), T classification as indicated by Fig. 1f (p < 0.001), and lymph node metastasis as shown in Fig. 1g (p < 0.001). These results, therefore, suggested that the abundance of CAFs supported the progression of BLCA. These results were further verified by analyzing the role of CAFs in the GEO BLCA cohort. Similar results were obtained since CAFs were highly abundant in the TME, showed a strong correlation with the ESTIMATE score (Fig. 2a), and lowered the Overall Survival (OS) of BLCA patients (Fig. 2b). Moreover, CAFs were closely associated with the stage of BLCA in patients and lymph node metastasis (Fig. 2c), which strongly validated the findings from TCGA. Although CAFs had no significant effect on distant metastasis in BLCA in both cohorts due to the limited number of M1 patients, the study still observed a trend in which CAFs promoted distant metastasis. Therefore, the above results suggested that the abundance of CAFs was a poor prognostic factor and enhances the progression of BLCA.

### Identification of 74 hub genes related to CAFs as well as their enriched functions and pathways

**Fig. 3:**
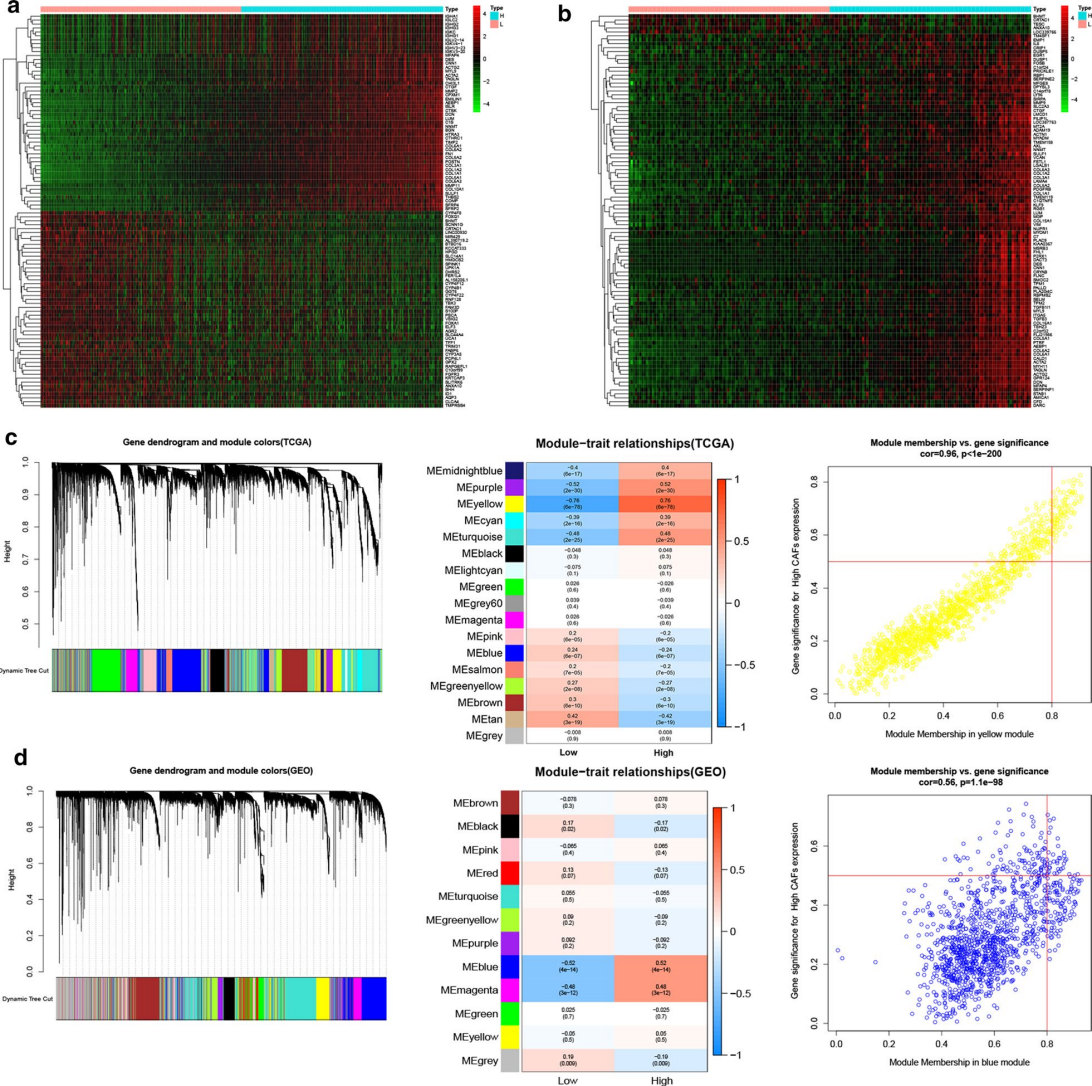
DEGs between high and low CAFs groups and significant CAFs related modules. **a–b ** The top 50 DEGs between high and low CAFs in TCGA (**a**) and GEO (**b**). **c–d** CAFs related modules in TCGA (**c**) and GEO (**d**). The yellow module in TCGA and the blue module in GEO showed the closest relationship with CAFs

We further categorized patients into the high and low CAFs groups then screened for DEGs in the TCGA and GEO cohorts. A total of 555 and 187 genes were differentially expressed between the low and high CAFs groups in the TCGA and GEO cohorts. The heatmap shows the top 50 DEGs in Fig. 3a, b. Moreover, WCGNA was applied to screen for modules that had the most significant association with levels of CAFs in both the TCGA (Fig. 3c) and GEO cohorts (Fig. 3d). The yellow module in the TCGA cohort showed the most significant association with a correlation level of 0.76, while the correlation between gene significance (GS) and module membership (MM) was 0.96. Similarly, the correlation between the blue module and the abundance of CAFs was shown to be 0.52, while GS and MM’s correlation was 0.56.

**Fig. 4:**
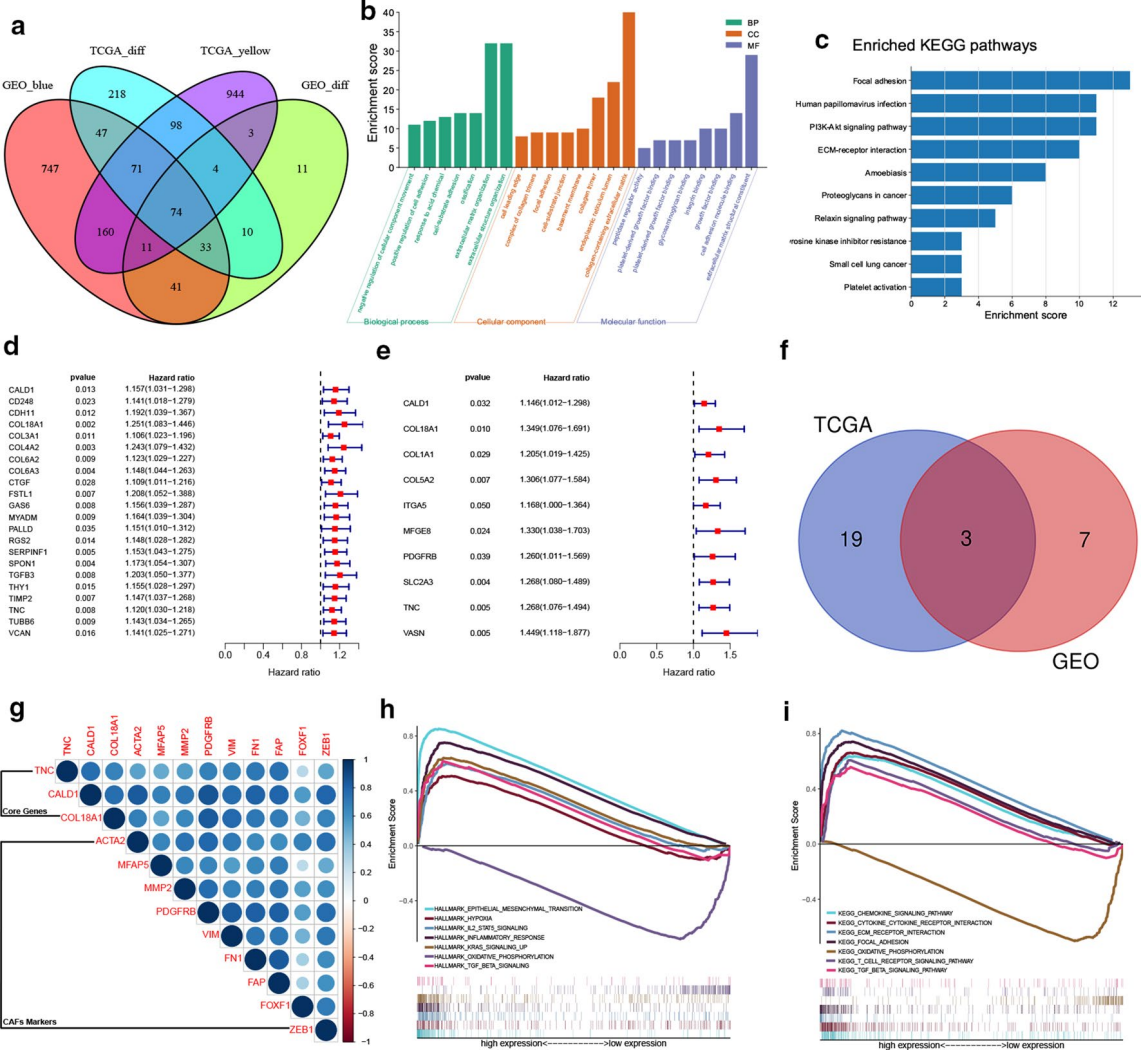
Screening of key CAFs-related genes in TCGA and GEO cohorts. **a–b** The intersection of DEGs and genes from the most related modules in TCGA and GEO cohorts revealed 74 hub genes closely related to CAFs abundance. **b** GO functional analysis of the 74 genes. **c** KEGG enrichment pathways of the 74 genes. **d-e** Survival-related genes by univariate Cox regression of the 74 gens in TCGA (D) and GEO (E) cohorts, genes with p-value less than 0.05 were shown in the forest plot. **f** The intersection of survival-related hub genes in TCGA and GEO revealed CALD1, COL18A1 and TNC as three key genes. **g** Correlation of the three key genes with CAFs markers in TCGA. **h–i** GSEA results of the Hallmark (**h**) and C2 Kegg gene sets (**i**) for high CALD1 expression group. The high CALD1 expression group was critically involved in the processes modulating the TME, including EMT, hypoxia, extracellular matrix remodeling and cytokine regulation, as revealed by GSEA

Additionally, the intersection of DEGs and genes in the most related modules identified 74 hub genes (Fig. 4a). Go functional analysis and KEGG enrichment analysis indicated that these genes were crucial in functions related to remodeling of the extracellular matrix. Notably, the following GO terms were enriched; extra matrix organization, collagen-containing extracellular matrix and extracellular matrix structure constituent, et al. (Fig. 4b). On the other hand, the following KEGG pathways were enriched; focal adhesion, ECM-receptor interaction, et al. (Fig. 4c).

###  Identification of three key genes related to CAFs in BLCA

Univariate cox regression was first conducted on both the TCGA and GEO cohorts based on hub genes’ expression. The results showed that 22 and 10 genes, respectively, were significantly related to patients’ survival with p values less than 0.05. The genes’ p values and hazard ratios were shown in forest plots separately (Fig. 4d, e). The intersection of survival-related hub genes in TCGA and GEO identified CALD1, COL18A1 and TNC as the three key genes related to CAFs and further influenced OS in BLCA (Fig. 4f). Notably, all these genes were significantly correlated with markers of CAFs, including; ACTA2 (α-SMA), MFAP5, MMP2, PDGFRB, VIM, FN1, FAP, FOXF1 and ZEB1 (Fig. 4g) [16]. Additionally, TNC was reported to be a biomarker of CAFs [15] and is a well-known independent risk factor for BLCA [17]. COL18A1 was previously reported to be involved in a 12-gene progression score significantly associated with progression[18]. CALD1 was also defined as a poor prognostic factor in BLCA [19]. In the present study, we selected CALD1 for further analysis. GESA analysis through the hallmarks gene sets confirmed that CALD1 was positively involved in pathways related to epithelium to mesenchymal transition and hypoxia, which are crucial for inducing immunosuppression of the TME (Fig. 4h). Besides, GSEA of KEGG pathways indicated that CALD1 was involved in multiple microenvironment remodeling pathways such as adhesion molecules cams, ECM receptor interaction and focal adhesion. It was also enriched in immune-related pathways, including the chemokine signaling pathway and cytokine-cytokine receptor interaction (Fig. 4I).

### Correlation between CALD1, OS, and clinical characteristics in the TCGA BLCA cohort and its involvement in the modulation of the TME

**Fig. 5:**
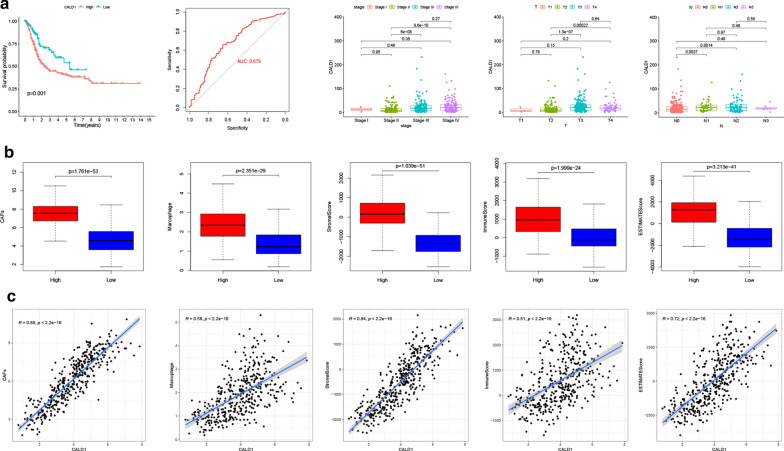
CALD1 regulated the TME and promoted the progression of BLCA. **a** Kaplan-Meier curves of OS between high and low CALD1 group using Log-Rank test in TCGA BLCA cohort. ROC curve demonstrated the accuracy of CALD1 in predicting cancer progression, with an AUC of 0.679. The expression of CALD1 was significantly elevated with cancer progression. Three different bar plots showed the expression level of CALD1 in different tumor stages, T and N classification, respectively. **b** CAFs (p < 0.001), macrophages (p < 0.001), stromal score (p < 0.001), immune score (p < 0.001) and ESTIMATE score (p < 0.001) in the CALD1 high expression group were significantly higher than those in the CALD1 low expression group. **c** Close relationships of CALD1 with CAFs (R = 0.88, p < 0.001), Macrophages (R = 0.58, p < 0.001), stromal score (R = 0.84, p < 0.001), immune score (R = 0.51, p < 0.001) and ESTIMATE score (R = 0.72, p < 0.001) were observed in the TCGA cohort

In the TCGA BLCA cohort, CALD1 was shown to markedly impact BLCA patients’ OS since there was a significant difference between the high and low CALD1 expression groups (p = 0.001). Additionally, the predictive value of CALD1 in cancer progression was confirmed through the ROC curve with an AUC of 0.679. Moreover, the expression levels of CALD1 differed significantly between different stages, T and N classifications (Fig. 5a). Furthermore, the study observed a trend of increasing CALD1 level with cancer metastasis, although no statistical significance was obtained. We further compared CAFs, macrophages and ESTIMATE scores between the high and low CALD1 expression groups. Results showed that the high CALD1 group had significantly higher CAFs, macrophages, stromal, immune, and ESTIMATE scores than the low CALD1 group (Fig. 5b, c). These results, therefore, indicated that CALD1 was a detrimental factor in the progression of BLCA. The findings also confirmed that CALD1 was involved in modulating both stromal and immune microenvironment, which was possibly achieved through CAFs and macrophages.

### Involvement of CALD1 in the regulation of TIICs and the immune checkpoint pathway

**Fig. 6:**
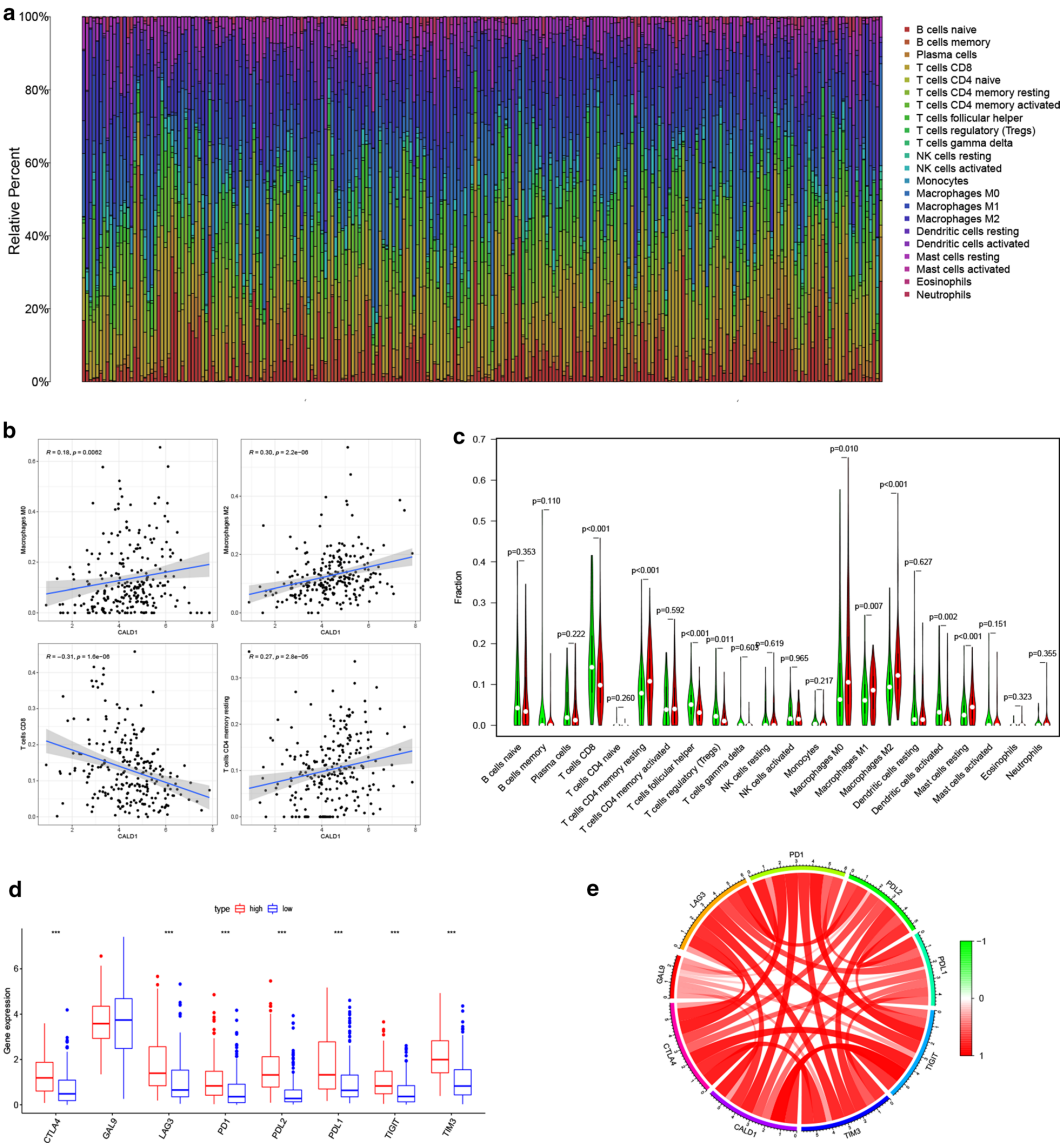
CALD1 was significantly associated with a variety of TIICs and immune checkpoint related molecules. **a **The relative percentage of 22 kinds of TIICs calculated by the CIBERSORT algorithm was shown in the bar plot. **b** CALD1 was positively correlated with macrophages M0 (R = 0.18, p = 0.006), macrophages M2 (R = 0.30, p < 0.001), T cells CD4 memory resting (R = 0.27, p < 0.001), and negatively correlated with T cells CD8 (R=-0.31, p < 0.001). **c** The difference in infiltrated levels of TIICs between low and high CALD1 expression groups. **d **Higher expression levels of CTLA4 (p < 0.001), LAG3 (p < 0.001), PD-1 (p < 0.001), PD-L1 (p < 0.001), PD-L2 (p < 0.001), TIGIT (p < 0.001) and TIM3 (p < 0.001) were observed in the high CALD1 group compared with low CALD1group.  **e**Close relationships between CALD1 and immune checkpoint-related genes in the TCGA cohort

The CIBERSORT algorithm was further used to validate the correlation of CALD1 with TIICs in BLCA. The proportion of each TCGA BLCA patient’s TIICs was analyzed using the CIBERSORT algorithm (Fig. 6a). Notably, correlation analysis showed that CALD1 was positively associated with macrophages (M0, M2) and negatively related to CD8 + T cells (Fig. 6b). A comparison of the TIICs levels between the high and low expression of CALD1 also confirmed an elevated level of macrophages (M0, M2) and decreased CD8 + T cells in the high CALD1 expression group (Fig. 6c). Consequently, the study further examined whether CALD1 was correlated with immune checkpoints such as PD-L1, which was also crucial in predicting immunotherapy efficacy in BLCA. Immune-checkpoint-related genes, including CTLA-4, LGALS9 (GAL9), LAG-3, PDCD1 (PD-1), PDCD1LG2 (PD-L2), CD274 (PD-L1), TIGIT and HAVCR2 (TIM-3), were therefore selected for further analysis. Interestingly, almost all the genes (CTLA-4, LAG-3, PD1, PDL2, PDL1, TIGIT and TIM-3) were up-regulated in patients with high expression of CALD1 (Fig. 6d, e). These results, therefore, highlighted the role of CALD1 in regulating TIICs and immune checkpoint pathways.

### Validation of the immune regulatory role of CALD1 in the GEO cohort

**Fig. 7:**
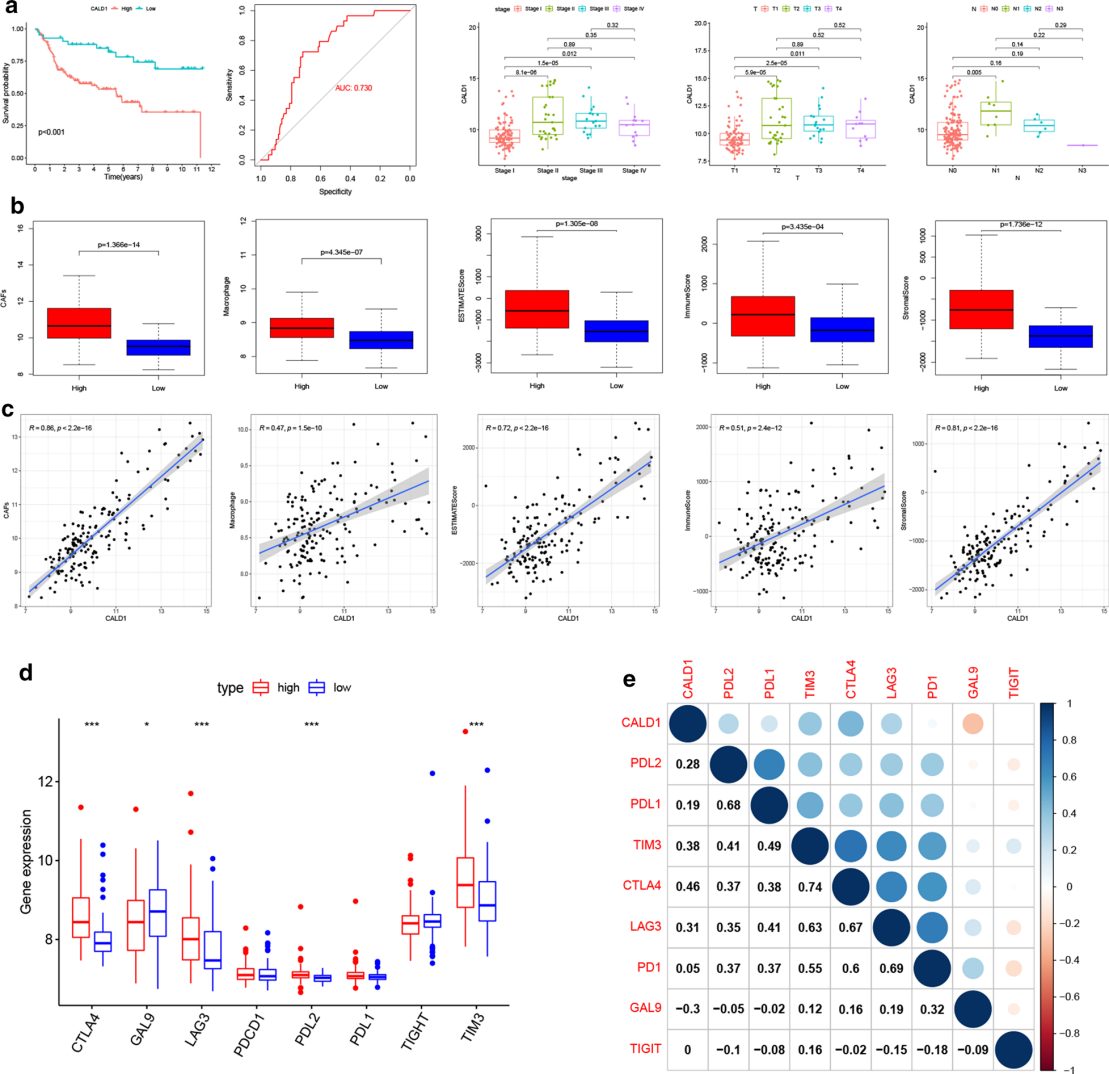
Validation of the TME regulating, Oncogenic promoting and immune checkpoint associating role of CALD1 in the GEO cohort. **a **Kaplan-Meier curve validated the difference of OS between high and low CALD1 expression BLCA Patients in the GEO cohort, with a P-value < 0.001. The AUC value of the ROC curve for CALD1 prediction of tumor progression in the GEO cohort was 0.730. A significantly higher level of CALD1 was also detected in patients with higher grade, stage, T and N classification in the GEO cohort. **b-c** CAFs (p < 0.001), macrophage (p < 0.001), stromal score (p < 0.001), immune score (p < 0.001) and ESTIMATE score (p < 0.001) were significantly different between the high and low CALD1 expression groups, and their expression level was also strongly correlated with CALD1(CAFs: R = 0.86, p<0.001; macrophage: R = 0.47, p < 0.0011; stromal score: R = 0.72, p< 0.001; immune score: R = 0.51, p< 0.001; stromal score: R = 0.81, p<0.001). **d-e** Close relationships were validated between CALD1 and immune checkpoint related genes, including CTLA4 (p < 0.001, R = 0.46), GAL9 (p<0.05, R=−0.30), LAG3 (p<0.001, R = 0.31), PD-L2 (p<0.001, R = 0.28) and TIM3 (p<0.001, R = 0.38) in the GEO cohort

To validate the results from the TCGA cohort, we further analyzed the effect of CALD1 on BLCA prognosis in the GEO cohort. The results showed that high expression of CALD1 was significantly associated with a shorter OS. Moreover, the ROC curve revealed that CALD1 had an AUC of 0.730 in predicting localized BLCA progression to metastatic BLCA. Significant differences in the expression levels of CALD1 were also observed between stage as well as T and N classification (Fig. 7a). Moreover, up-regulated CAFs, macrophages and ESTIMATE scores were shown in the high CALD1 expression group compared to the low CALD1 expression group (Fig. 7b, c). Furthermore, significantly higher CTLA4, LAG3, PDL2, TIM3 and lower GAL9 expression were observed in patients with high expression of CALD1 (Fig. 7d, e), further confirming the role of CALD1 in regulating immune checkpoints.

### Expression of CALD1 in clinical specimens in the validation cohort

**Fig. 8:**
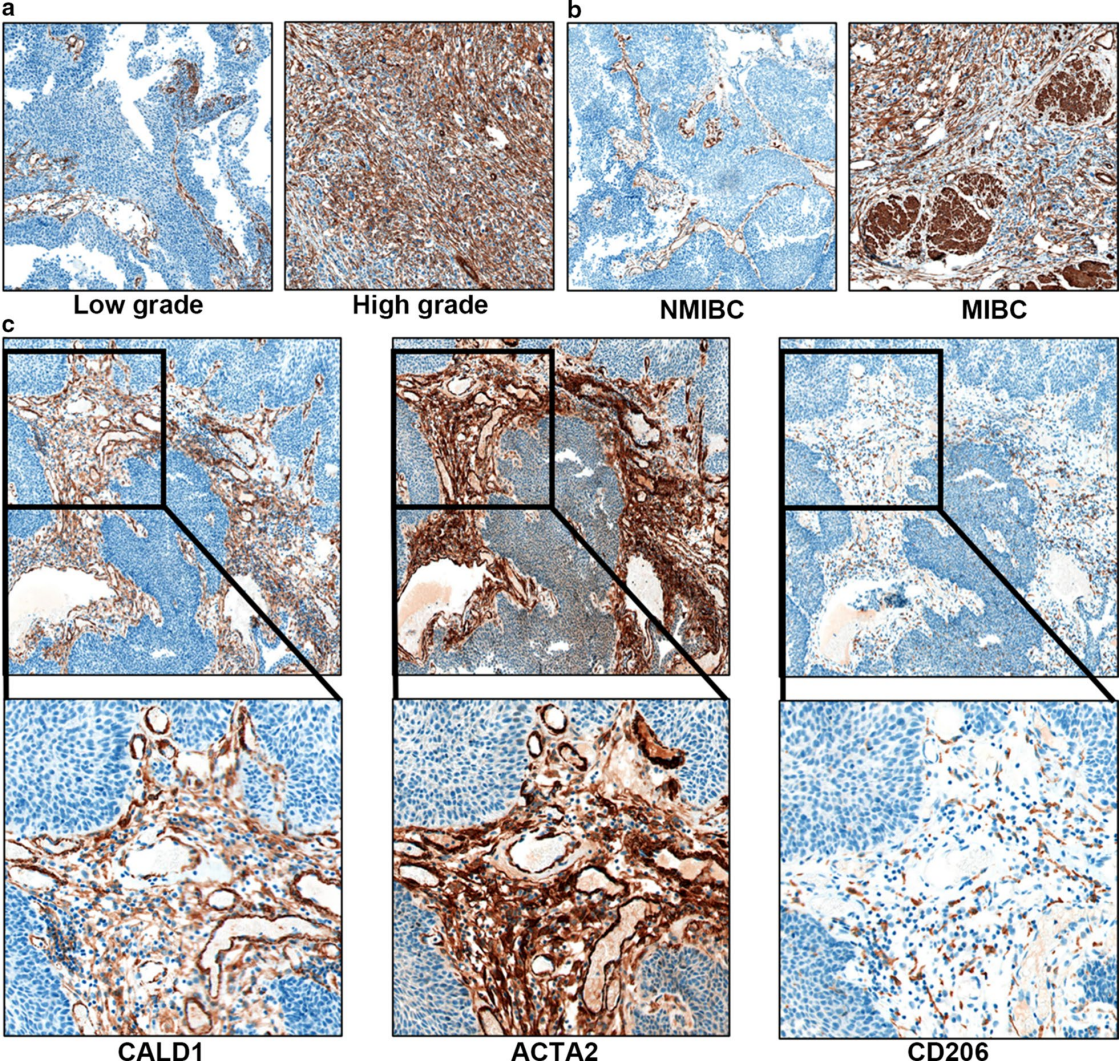
Verification of the TME regulating and cancer-promoting role of CALD1 in clinical samples. **b** CALD1 expression was significantly elevated in high-grade BLCA samples. **c **High co-expression of CALD1 and ACTA2 was observed in multiple BLCA samples, highlighting the CAFs associated role of CALD1. CD206 expressed in the regions with high CALD1 and ACTA2, which verified the close relationship between CALD1, CAFs, and macrophages M2, suggesting the regulatory effect of CALD1 on both stromal and immune components of the TME

40 BLCA patients with different grades, stages and TNM classifications were recruited to validate the above results. Expression levels of CALD1 were examined in pathological sections after clinical treatment with TURBT or radical cystectomy. The results revealed high expression levels of CALD1 in patients with a higher grade (Fig. 8a) and stage (Fig. 8b). Moreover, co-expression was found between CALD1, ACTA2 and CD206 in the tumor stroma, especially in patients with advanced BLCA (Fig. 8c). The IHC score result further confirmed the correlation of CALD1 with ACTA2 (p < 0.001) and CD206 (p < 0.001) (Table 2). These results confirmed the association of CALD1 with CAFs and macrophages, which may further lead to the progression of BLCA.

**Table 2:** Correlation of CALD1 with CD206 and ACTA2 in clinical samples

Variable	N	CALD1(+) n(%)	CALD1(−) n(%)	P-value (Fisher exact test)
CD206				< 0.001
+	20	19 (95 %)	1 (5 %)	
-	20	7 (35 %)	13 (65 %)	
ACTA2				< 0.001
+	26	23 (88.5 %)	3 (11.5 %)	
-	14	3 (21.5 %)	11(78.5 %)	

## Discussion

BLCA is among the cancers characterized by infiltration of abundant immune cells in the TME, confirmed by BCG’s treatment efficiency. Moreover, recent advances in immune checkpoint inhibitor therapy for BLCA further demonstrate that BLCA is profoundly regulated by tumor immunity [20].

Tumor cells and the microenvironment are a whole functional unit where the cells are regarded as seeds, and the microenvironment is considered the soil [21]. Therefore, tumor cells and the microenvironment interact with each other and evolve together to promote tumors. Moreover, the TME is a complex integrated system divided into the immune microenvironment and non-immune microenvironment. Stromal components dominated the non-immune microenvironment [22], especially CAFs [23]. Emerging evidence confirmed the crosstalk between stromal and immune components of the TME [24, 25]. Among the immune components, macrophages are usually the most abundant TIICs in the tumor microenvironment, including in BLCA[26]. Macrophages consist of two groups with different phenotypes, namely M1 and M2. Macrophages M2 are associated with immunosuppressive functions, angiogenesis, and the extracellular matrix’s degradation, contributing to cancer migration and metastasis [27].

Numerous studies have demonstrated that the interaction between CAFs and macrophages can further promote the progression of cancer. For instance, Mazur et al. reported that CAFs could increase macrophages’ adhesive ability and promote cancer invasion and metastasis [28]. Additionally, Betul et al. showed that CAFs could recruit and differentiate monocytes into M2 macrophages and exert their immunosuppressive role through the PD-1 axis [29]. The macrophages could also reversely modulate the status of CAFs. Zhang et al. demonstrated that macrophages could turn umbilical cord mesenchymal stem cells into CAFs, further promoting cancer progression through Epithelial-mesenchymal Transition (EMT) [30]. The above evidence demonstrates that CAFs are involved in tumor immunomodulation and promoted tumor progression by interacting with macrophages. In the present study, CAFs were shown to be abundant in BLCA. Moreover, our results also indicated that CAFs conferred significant adverse effects on the progression of BLCA and OS. We also confirmed a close relationship between CAFs and macrophages. Therefore, combined with existing evidence, the interaction between these two cell types can significantly promote the progression of BLCA. Targeting these two cell types may be a potential strategy for the treatment of BLCA.

On the other hand, we identified three key genes related to CAFs by WCGNA and took CALD1 for further study. Previous researches have demonstrated that CALD1 was involved in cell proliferation and migration through actin cytoskeleton recombination [31]. It was also recognized as a tumor-specific splicing variant in colon, bladder, and prostate tissue samples. The mis-splicing of CALD1 was an independent epigenetic event that was related to the destruction of the tight junctions between epithelial cells, hence altering the stiffness of the extracellular matrix and promoting cancer invasion and metastasis [32]. Studies also confirmed CALD1 to be a risk factor for the progression of BLCA, but its role with regard to CAFs and immune regulation in BLCA is yet to be reported.

In this study, the vital role of CALD1 in the progression of BLCA was demonstrated in three independent cohorts. As a key gene associated with CAFs, CALD1 showed strong correlations with stromal and immune scores, suggesting its dual regulation of stromal and immune components. Also, CALD1 exhibited essential involvements in the processes modulating the TME, including EMT [33], hypoxia [34], extracellular matrix remodeling [35] and cytokine regulation [36], as revealed by GSEA. Moreover, CALD1 represented a positive correlation with M2 macrophages and a negative correlation with CD8 + T cells. Also, high correlations between the expression levels of CALD1 and multiple immune checkpoint genes were observed. These results highlighted the critical function of CALD1 in inducing immunosuppression in BLCA. The increased expression of CALD1 may be correlated with high expression of macrophage M2 and checkpoints and the depletion of T cell CD8. In the clinical validation cohort, we well confirmed the co-expression of CALD1, ACTA2 and CD206 through immunohistochemistry, and the IHC score further demonstrated the close correlation of CALD1 with CAFs and macrophages. At the same time, in tumor specimens with different stages and depth of tumor invasion, we found significantly differentially expressed CALD1 level, which further indicated that CALD1 has the potential to be used as a marker of BLCA progression.

From our study, we confirmed that CALD1 was a risk factor in the progression of BLCA. It was also approved for the first time that CALD1 regulated the tumor microenvironment associating with CAFs and macrophages. Also, our results clearly demonstrated the importance of bioinformatics analysis in cancer researches. Through the bioinformatics means such as WCGNA, CIBERSORT and MCP-counter, combined with clinical verification, we can get information that other means cannot achieve. It is believed that with the continuous progress of the algorithms, bioinformatics can provide more significant help for clinical diagnosis and treatment.

Despite the insightful findings, limitations still exist in our study. First, relationships among CALD1, CAFs, macrophages and immunosuppression were only verified by correlation analysis. Further verification from *in vitro* and *in vivo* experiments, including single-cell RNA sequencing, is required for exploring the exact mechanisms. Second, the potential of CALD1 to become a specific marker of CAFs in BLCA still need validation since we did not discuss its expression in normal fibroblasts. Last, although significant co-expression was found between CALD1, ACTA2 and CD206 in BLCA sections, a more extensive validation cohort is still necessary to avoid the selection bias.

## Conclusions

In conclusion, this study confirmed the pro-tumor function of CAFs and identified CAFs-related genes in BLCA through WCGNA and screening for DEGs in both the TCGA and GEO cohorts. Moreover, CALD1 was recognized as one of the key genes related to CAFs and outcomes in BLCA. Further analysis showed that CALD1 played a vital role in regulating the TME of BLCA. Furthermore, CIBERSORT and correlation analysis confirmed that CALD1 was related to the infiltration level of multiple TIICs in the TME, especially macrophages M2 and CD8 T cells. It was also shown that high expression of CALD1 might lead to an increased level of immune checkpoint-related genes, including PD-L1. Therefore, CALD1 may be associated with the immunosuppression status of TME in BLCA, which further leads to tumor progression. Further studies on CALD1 may provide insights into the immune network in BLCA and offer new targets for cancer treatment.

## Data Availability

The datasets used and analyzed during the current study are available from Gene Expression Omnibus (http://www.ncbi.nlm.nih.gov/geo/) and The Cancer Genome Atlas (portal.gdc.cancer.gov). All the results will be available at reasonable request. We would like to thank Professor Chen Jing and Dr. Jin Hong from The Second Affiliated Hospital of Soochow University for their generous help in this study.

## Abbreviations

CAFs: Cancer-Associated Fibroblasts
TAMs: Tumor-Associated Macrophages
WGCNA: Weighted Gene Co-expression Network Analysis
TCGA: The Cancer Genome Atlas
GEO: Gene Expression Omnibus
NMIBC: Non-Muscle Invasive Bladder Cancer
MIBC: Muscle Invasive Bladder Cancer
DEGs: Differentially Expressed Genes
GSEA: Gene Set Enrichment Analysis
GO: Gene Ontology
KEGG: Kyoto Encyclopedia of Genes and Genomes
OS: Overall survival
EMT: Epithelial-mesenchymal Transition
